# Genetic Resistance to *Mycobacterium tuberculosis* Infection and Disease

**DOI:** 10.3389/fimmu.2018.02219

**Published:** 2018-09-27

**Authors:** Marlo Möller, Craig J. Kinnear, Marianna Orlova, Elouise E. Kroon, Paul D. van Helden, Erwin Schurr, Eileen G. Hoal

**Affiliations:** ^1^Division of Molecular Biology and Human Genetics, Faculty of Medicine and Health Sciences, DST-NRF Centre of Excellence for Biomedical Tuberculosis Research, South African Medical Research Council Centre for Tuberculosis Research, Stellenbosch University, Cape Town, South Africa; ^2^Program in Infectious Diseases and Immunity in Global Health, The Research Institute of the McGill University Health Centre, Montreal, QC, Canada; ^3^McGill International TB Centre, McGill University, Montreal, QC, Canada; ^4^Departments of Medicine and Human Genetics, McGill University, Montreal, QC, Canada

**Keywords:** host genetics, resistance, tuberculosis, resisters, susceptibility

## Abstract

Natural history studies of tuberculosis (TB) have revealed a spectrum of clinical outcomes after exposure to *Mycobacterium tuberculosi*s, the cause of TB. Not all individuals exposed to the bacterium will become diseased and depending on the infection pressure, many will remain infection-free. Intriguingly, complete resistance to infection is observed in some individuals (termed resisters) after intense, continuing *M. tuberculosis* exposure. After successful infection, the majority of individuals will develop latent TB infection (LTBI). This infection state is currently (and perhaps imperfectly) defined by the presence of a positive tuberculin skin test (TST) and/or interferon gamma release assay (IGRA), but no detectable clinical disease symptoms. The majority of healthy individuals with LTBI are resistant to clinical TB, indicating that infection is remarkably well-contained in these non-progressors. The remaining 5–15% of LTBI positive individuals will progress to active TB. Epidemiological investigations have indicated that the host genetic component contributes to these infection and disease phenotypes, influencing both susceptibility and resistance. Elucidating these genetic correlates is therefore a priority as it may translate to new interventions to prevent, diagnose or treat TB. The most successful approaches in resistance/susceptibility investigation have focused on specific infection and disease phenotypes and the resister phenotype may hold the key to the discovery of actionable genetic variants in TB infection and disease. This review will not only discuss lessons from epidemiological studies, but will also focus on the contribution of epidemiology and functional genetics to human genetic resistance to *M. tuberculosis* infection and disease.

## Introduction

**Figure 1:**
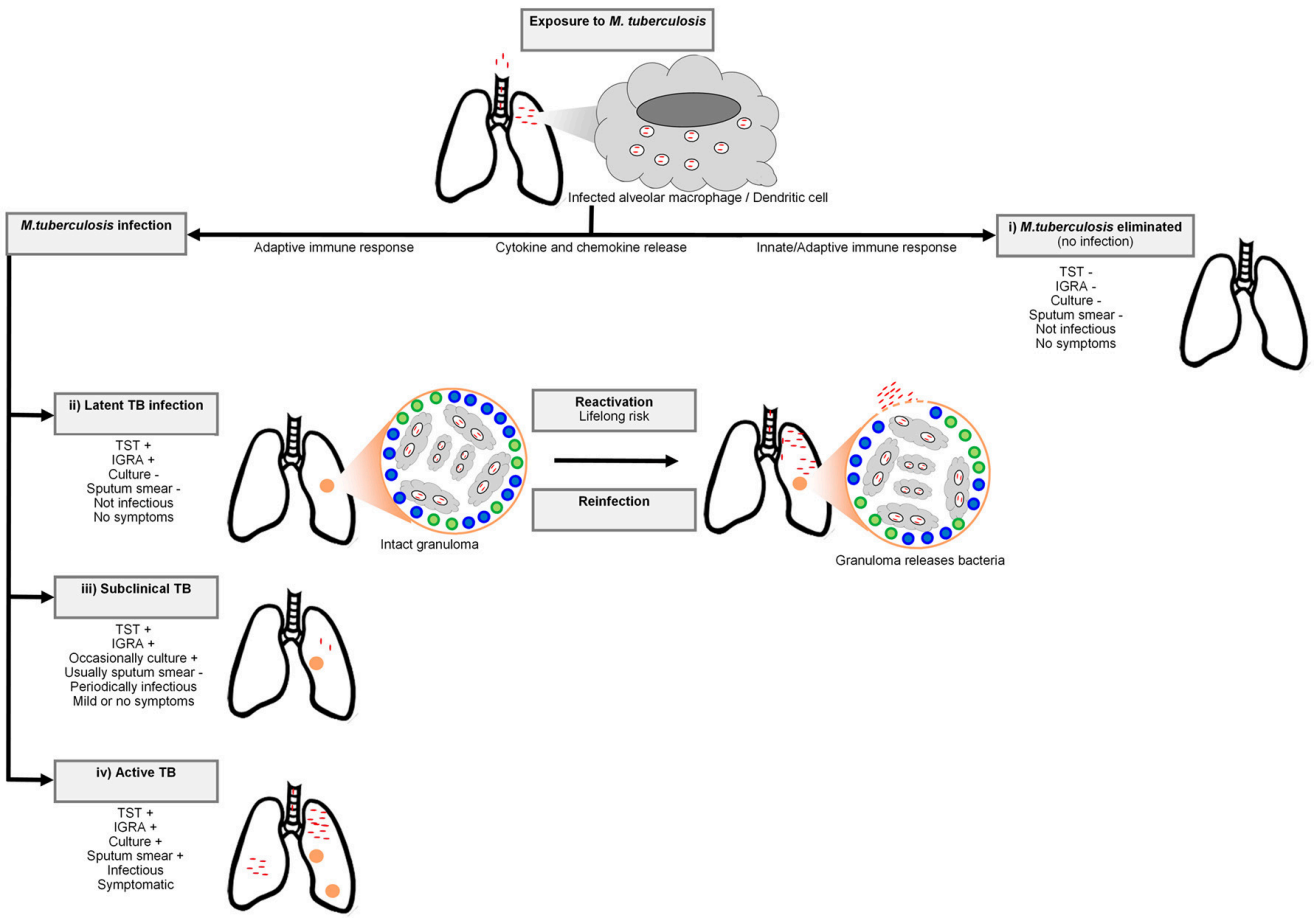
A simplified representation of the *Mycobacterium tuberculosis* infection spectrum and outcomes. The bacteria enter the respiratory system of the host via inhaled droplets and are engulfed by macrophages and dendritic cells. There are four potential outcomes after bacterial inhalation: (i) *M. tuberculosis* is immediately eliminated by the pulmonary immune system, (ii) the bacteria are contained in granulomas by recruited adaptive immune cells (including T cells and B cells) and infection does not progress to active TB. Although this containment can last for a lifetime, *M. tuberculosis* can also disseminate from granulomas (reactivation) or reinfection with another mycobacterial strain can occur, resulting in active TB, (iii) sub-clinical disease characterized by intermittent symptoms and periodic infectiousness, or (iv) infection develops into active TB. Adapted from Pai et al. (3) and Möller et al. (4).

Tuberculosis (TB), caused by the human pathogen *Mycobacterium tuberculosis*, was the leading cause of death due to a single infectious agent in 2016, resulting in 1.6 million deaths (1). The bacterium is spread through the air by droplet nuclei containing *M. tuberculosis* from the lungs of individuals with active disease to the respiratory tract of uninfected individuals (2). Infection by *M. tuberculosis* is a complex, multistage process progressing from the first encounter with the bacterium (Figure 1). For this reason a multistep course of disease has to be imagined (5). After inhalation, the droplet nuclei move to the alveoli where the bacteria are phagocytosed by alveolar macrophages and dendritic cells. The phagocytosis of the bacterium invokes a strong host cellular immune response and a cascade of events is triggered that involves cytokines and chemokines (2). Not all individuals exposed to the bacterium will become infected and depending on the infection pressure, many will remain free of infection. In infected individuals the bacteria will begin to replicate in the intracellular environment and migrate to lymph nodes in the lung through the lymphatic system (6). In the first 2–8 weeks after infection, cell-mediated immunity will develop (7) and conversion to tuberculin reactivity takes place (6). To limit the spread and replication of the bacteria, granulomas are formed by activated T lymphocytes and macrophages. The majority of individuals will remain asymptomatic and contain the bacterium, and enter a stage termed latent TB infection (LTBI). Remarkably, it is estimated that ~25% of the global population was latently infected with *M. tuberculosis* in 2014 (8). At this stage the immune system can contain the infection, but if it fails, the infection may progress to active disease (7). Only 5-15% of immunocompetent LTBI positive individuals will progress to clinical TB (8). In these cases, the bacteria continue to replicate and disease symptoms will start to appear. Common symptoms of TB include persistent coughing, fever, coughing of blood, night sweats, weight loss, and chest pain. Diagnosis of pulmonary TB is possible through smear microscopy, bacterial culture of sputum or GeneXpert (9).

LTBI is at present inferred from measures of acquired anti-mycobacterial immunity, such as a tuberculin skin test (TST) and/or interferon gamma release assay (IGRA). The TST was the original gold standard for LTBI diagnosis (10). A delayed hypersensitivity reaction to mycobacterial antigens is measured by injecting tuberculin purified protein derivative (PPD) intradermally into the forearm, followed by measuring the induration 48 h later (10). A positive TST in an immunocompetent individual is defined as an induration of 10 or more millimeters in high prevalence countries. The PPD antigens are not specific to *M. tuberculosis* and may result in false positive reactions if individuals were exposed to non-tuberculous mycobacteria or immunized with *M. bovis BCG* (10). In contrast, IGRA is a whole blood assay, which uses the specific *M. tuberculosis* antigens ESAT-6, CFP-10, & TB7.7 to stimulate antigen-specific CD4 T cells to release interferon gamma, which is then measured. Even within LTBI individuals there is a spectrum of infection states ranging from the early elimination of infection to subclinical TB, which cannot be differentiated by TST or IGRA (11–16).

In the case of immediate bacterial clearance, or complete resistance to infection (observed in a small fraction of the population) the innate immune system will inactivate the bacteria at the site of infection without the stimulation of an acquired immune response. These individuals, recently labeled innate resisters by Simmons et al. will have continued negative TST or IGRA results despite heavy and continued exposure to *M. tuberculosis* and will not be at risk of clinical TB (11, 17). The resister phenotype is likely heterogeneous and could include individuals who mount a protective adaptive immune response (termed adaptive resisters) perhaps involving B cells or unconventional T cell responses during early clearance of the bacterium (17). Also of interest are those LTBI individuals who have no risk of progression to clinical TB, labeled non-progressors, possibly due to an exceptionally well-contained infection or absence of viable bacteria in the granuloma (18). The elucidation of the genetic correlates that contribute to these infection and disease resistance phenotypes is a priority as it may translate to new interventions to prevent, diagnose, or treat TB.

Genetic investigations of TB susceptibility have been ongoing for decades, but gained momentum in recent years due to the availability of improved methodological approaches and technological advances. The majority of studies made use of classical approaches employed by clinical genetics and genetic epidemiology (linkage and association studies), but these have encountered difficulties also faced by genetic investigations of other complex diseases. Three continuing challenges involve polygenicity, the definition of TB phenotypes and the collection of appropriately large study cohorts with carefully defined homogenous phenotypes (11, 19, 20). More recently functional genetic studies, including epigenetics, microRNAs and transcriptomics, have also shed light on the genetic basis of TB susceptibility (21). This review will discuss the early epidemiological evidence of genetic susceptibility to *M. tuberculosis* infection and disease progression, but will also focus on the contribution of genetic epidemiology and functional genetics while highlighting controversies, current research gaps, and future developments.

## Epidemiological evidence of genetic susceptibility to *M. tuberculosis* infection and clinical TB resistance

Epidemiological evidence confirms the presence of both resisters and non-progressor phenotypes in high exposure settings. Approximately 50% of close household contacts develop positive TST or IGRA tests (22–26) and there are multiple examples of homogenous high exposure and heterogeneous infection [reviewed by Verrall et al. (27)]. For example the HIV epidemic resulted in TB control failure in South African mines during the 1990s with notification results exceeding 4,000 per 100 000 person-year (28). In the midst of this perfect TB storm, 13% of HIV negative miners had TST = 0 mm responses (28). TB outbreaks onboard ships of the United States Navy yielded similar findings with 5–10% of crew members at highest risk of exposure remaining TST negative (29, 30). Likely resisters were also detected amongst nurses who were exposed to TB patients (31–34). The contribution of hereditary factors to clinical disease susceptibility was recognized even before the discovery of the bacterium, due to the observation that TB often occurred in several individuals from the same family (35). Koch's discovery of the bacterium in 1882 meant that it would be several decades before the host genetic component would again be considered as a contributing factor to disease. In 1943 a seminal study investigating TB concordance found that monozygous twins were significantly more likely to both become diseased than dizygous twins (66.7 vs. 23%) (36). The study also included relatives of the twins and found that the degree of relatedness to the TB index case determined the risk of developing active disease (36). Descriptions of the natural history of clinical TB from the era before antibiotic treatment provide valuable insights into resistance to active disease. For example, pulmonary TB in immunocompetent individuals with no antimicrobial chemotherapy was fatal in ~50% of cases; 25% of individuals remained ill with chronic TB and the remaining 25% spontaneously achieved cure (37).

In addition to these “experiments of nature,” animal models have provided important evidence for a contribution of host genetics to TB infection and progression to clinical disease. Different patterns of disease resistance after infection have been observed in guinea pigs and inbred strains of mice (38). The rabbit model was extensively used by Lurie et al. to study resistance to disease progression and clearly represented two forms of genetically controlled resistance (39–41). The so-called “resistant” rabbits survived approximately twice as long as susceptible rabbits. Susceptible rabbits developed disseminated disease, while resistant rabbits developed cavitary TB (40, 42). An additional evaluation of Lurie's findings by Werneck-Barroso indicated that 20–40% of rabbits exposed to the bacterium did not develop disease and the majority of these did not become TST positive, even after prolonged exposure (43). This suggests that some animals had an intrinsic ability to resist natural *M. tuberculosis* infection and that the bacteria were eliminated without stimulating an acquired immune response (43).

No review of TB susceptibility would be complete without a discussion of the Lübeck disaster. During this tragedy, which took place from December 1929, 251 infants over a time period of 4 months were vaccinated with BCG accidentally contaminated with varying amounts of virulent *M. tuberculosis* [reviewed by Fox et al. (44)]. Clinical or radiological signs of TB were observed in 173 survivors, but 72 infants died from TB (44). Fox et al. pointed out three key lessons resulting from the accident. Firstly, 156 (68%) of those who had developed clinical disease, spontaneously resolved their symptoms, suggesting that newborn infants are remarkably resistant to TB. Secondly, based on available data, semiquantitative levels of *M. tuberculosis* contamination were inferred. At low levels of contamination, a wide range of clinical phenotypes was seen, revealing the extent of innate resistance to clinical TB. However, at high doses of *M. tuberculosis* contamination, most babies were susceptible to disease, indicating that extreme exposure will eventually overcome host innate (genetic) resistance to disease (44). Therefore, the dose of *M. tuberculosis* is key to determine TB outcome. Lastly, two infants received vaccines with the lowest levels of contamination, but quickly progressed to disease and death, perhaps indicating that they were most susceptible to TB (44).

Isolated populations with little or no known past exposure to the TB bacterium, such as the indigenous populations of the Americas and sub-Saharan Africa, have a significantly higher TB mortality than non-indigenous populations whose ancestors had a longer mycobacterial exposure time (45–47). These observations support the “virgin soil” hypothesis, which postulates that the previous lack of exposure to a pathogen leads to hyper-susceptibility to infection, morbidity and mortality. Newer DNA evidence indicates that the *M. tuberculosis* complex was in fact already widespread around 4000 years ago in Ethiopia and TB infection was also found in western-central and western African individuals who arrived in Brazil as slaves during 1769–1830 (48, 49). The introduction of (European) TB strains possibly exacerbated by local host genetic factors and poor living conditions, contributed to the high mortality observed in these “naïve” populations (50). For example, analyses of the indigenous population of Canada, limited to the Qu'Appelle Agency located in Southeastern Saskatchewan, indicated an annual TB mortality rate of 10% in 1890 which fell to 0.2% after 40 years, but half of the population was eradicated (45). This is suggestive of a strong selection for TB resistance genes. More recent examples of the introduction of *M. tuberculosis* to underexposed populations is that of the Northern Aché of eastern Paraguay and the Yanomami Indians of the Brazilian Amazon (47, 51). Prevalence (18.2%) and infection (64.6%) rates in the Northern Aché quickly rose within 6 years of the first detected TB case (51). TST anergy, possibly reflecting reduced cell-mediated immune responses, and increased antibody responses were common in individuals with active TB from both these populations (47, 51). This could indicate that there has been no selection for TB resistance mechanisms in these groups. In contrast, the decline in TB incidence in North America and Europe during 1830–950, before the introduction of antimicrobial chemotherapy, has been ascribed to the increase of genetically determined resistance to TB due to natural selection after years of mycobacterial exposure (35, 48, 52–55). However, an estimation using Swedish fertility and mortality data, which included age-specific pulmonary TB mortality, from 1891 to 1900 indicated that changes in only the genetic make-up of the population would have been unlikely to account for the extreme decline in TB mortality (56). Although surviving individuals had a fitness advantage of 7–15% per generation compared to individuals who died, statistical calculations indicated that selection would only have reduced the frequency of rare susceptibility variants if these variants had large effects. In contrast, if rare resistance variants were in fact rare, 300 years would not have been sufficient for selection to increase the frequency of these variants to epidemiologically significant frequencies. Despite this, evidence for the role of natural selection in TB resistance is bolstered by findings from population genetic studies of the immune system which provide a context for the genetic interface between humans and mycobacteria (57, 58, 59, 60, 61).

## Heritability, genetic epidemiology, and population genetics

Heritability, genetic epidemiology, and population genetic studies have made significant contributions to reveal the role of human genetic variation in susceptibility to TB infection. The investigations of TST and IGRA as quantitative traits have shown high heritability for both, conditional to *M. tuberculosis* exposure (TST above 50% and for IGRA between 30 and 50%). Heritability of quantitative TST reactivity (in mm) among young healthy children exposed to an active TB case was estimated at 92% in Chile (62). In the Gambia, the heritability of TST considered as a categorical trait and quantitative IGRA reactivity in healthy twins aged 12 to 83 years was estimated at 71 and 39%, respectively (63). In Colombia evidence was detected for a major co-dominant gene explaining ~65% of TST variability (64), and in a South African familial sample, the heritability of quantitative IGRA responses was estimated to be between 43 and 58%, depending on the nature of the stimulating antigen (65). Recent data from Uganda, carefully adjusted for shared environment, also detected significant heritability of interferon gamma in response to *M. tuberculosis* culture filtrate (23–35%), ESAT6 (15–48%), and Antigen 85B (11–34%) (66).

Only a few molecular studies have investigated the genetic factors underlying *M. tuberculosis* infection resistance using TST reactivity. Candidate gene association studies have focused on TST response as a binary trait according to various thresholds (0, 5, or 10 mm) with a weak association reported for interleukin 10 (*IL10)* promoter variants (67, 68). Increased IL-10 production may contribute to the suppression of adaptive immune responses (68). A candidate gene association study of autophagy-related genes and LTBI, defined by a TST response greater than 5 mm, identified an association between a non-coding Unc-51 Like Autophagy Activating Kinase 1 gene (*ULK1)* variant and LTBI (69). A possibly associated role for ULK1 in the regulation of TNF secretion, both non-specific and *M. tuberculosis*–induced autophagy, and *M. tuberculosis* replication in monocytes was established (69). A genome-wide association study (GWAS) of TST reactivity in HIV positive individuals from Tanzania and Uganda pinpointed a polymorphism on chromosome 5q31.1 that protected against *M. tuberculosis* infection (Table 1) (80). This variant is located near the gene encoding IL-9, which is produced by mast and Th2 cells during inflammatory responses and has been associated with bronchial responsiveness, possibly linking resistance against *M. tuberculosis* infection and airway inflammation (80). A GWAS in Iceland detected associations between TST positivity (induration size not specified) and HLA class II variants (Table 1) (79). An imputed GWAS was done in 4,426 cases with a self-reported positive TST (defined as the presence of an induration) and 84 290 controls selected from more than 200 000 23andMe participants with European ancestry who completed a questionnaire on infection history (81). The *HLA* rs2894257 variant on chromosome 6p21.32 was significantly associated with the presence of a TST induration (*p* = 8.16 × 10^−36^, OR 1.36, 95% CI 1.33–1.39) and then after further fine mapping of the locus multiple independent associations between a history of a positive TST and HLA were detected (Table 1) (81). The HLA class II region could contribute to infection resistance by reduced presentation of *M*. tuberculosis antigens to T cells (79). In Uganda, a genome-wide linkage analysis (GWLA) reported suggestive, but not significant, linkage of persistent TST negativity (defined as a TST < 10 or 5 mm according to age and HIV status) with chromosomal regions 2q21-2q24 and 5p13-5q22 (84). The chromosome 2q region was subsequently investigated using an association scan in two independent cohorts from Uganda and associations were found with variants in the Zinc finger E-box-binding homeobox 2 (*ZEB2*) and Glycosyltransferase Like Domain Containing 1 (*GTDC1*) genes (85). These variants may regulate the histone deacetylase pathway, which has been implicated in infection resistance by transcriptomic investigations (discussed below) (86). Two loci were identified by GWLA in an HIV negative population from South Africa (87). *TST1* was identified on chromosome 11p14 by focusing on the phenotype of TST > 0 mm vs. TST = 0 mm, and captures innate resistance to infection with *M. tuberculosis. TST2* was mapped to region 5p15 and influences the intensity of TST reactivity - captured as TST induration in mm. Hence, *TST2* reflects intensity of T-cell mediated anti-mycobacterial immune responses. The mapping of *TST1* has been confirmed in an independent sample of different ethnic origins in France, and it was also shown that *TST1* cannot be distinguished by linkage from *TNF1*, a locus controlling TNF production in response to BCG/IFN-gamma (88, 89).

**Table 1:** GWAS of TB infection and disease phenotypes. Adapted from Kinnear et al.(20).

**Population**	**Phenotype**	**Cases**	**Controls**	**Variant**	**Gene**	**Odds ratio [95% CI]**	**Reference**
Ghana	TB	921	1740	rs4331426	Gene desert (chromosome 18)	1.19 [1.13–1.27]	(70)
Gambia	–	1316	1382	–	–	–	–
USA	Extrapulmonary TB	48	57	rs4893980	*PDE11A*	0.13	(71)
–	–	–	–	rs10488286	*KCND2*	11.15	–
–	–	–	–	rs2026414	*PCDH15*	3.11	–
–	–	–	–	rs10487416	Unknown gene	5.56	–
Thailand	Young TB	433	295	rs6071980	*HSPEP1, MAFB* (intergenic, chromosome 20q12)	1.73 [1.42–2.11]	(72)
Japan	–	188	934	–	–	–	–
Indonesia	Pulmonary TB	108	115	rs2273061	*JAG1*	1.8 [1.18–2.72]	(73)
–	–	–	–	rs4461087	*DYNLRB2*	1.62 [1.1–2.37]	–
–	–	–	–	rs1051787	*EBF1*	0.57 [0.38–0.88]	–
–	–	–	–	rs10497744	*TMEFF2*	0.55 [0.38–0.82]	–
–	–	–	–	rs1020941	*TMEFF2*	0.57 [0.38–0.83]	–
				rs188872	*CCL17*	0.51 [0.33–0.78]	–
–	–	–	–	rs10245298	*HAUS6*	2.37 [1.09–5.16]	–
–	–	–	–	rs6985962	*PENK*	2.01 [1.12–3.61]	–
–	–	–	–	rs1418267	*ERP44*	3.19 [1.71–5.99]	–
Ghana	TB	2127	5636	rs2057178	*WT1* (intergenic)	0.77 [0.71–0.84]	(74)
Gambia	–	1207	1349	–	–	0.80 [0.70–0.91]	–
Russia	–	1025	983	–	–	0.91 [0.82–0.99]	–
Indonesia	–	4441	5874	–	–	0.84 [0.68–1.03]	–
South Africa	Pulmonary TB	642	91	rs2057178	*WT1* (intergenic)	0.62 [0.50–0.75]	(75)
–	–	–	–	rs11031728	*WT1* (intergenic)	0.61(0.50–0.75)	–
Russia	Pulmonary TB	5530	5607	rs4733781	*ASAP1*	0.84 [0.79–0.89]	(76)
–	–	–	–	rs10956514	*ASAP1*	0.85 [0.80–0.90]	–
–	–	–	–	rs1017281	*ASAP1*	0.85 [0.81–0.90]	–
–	–	–	–	rs1469288	*ASAP1*	0.84 [0.79–0.89]	–
–	–	–	–	rs17285138	*ASAP1*	0.85 [0.80–0.90]	–
–	–	–	–	rs2033059	*ASAP1*	0.83 [0.79–0.88]	–
–	–	–	–	rs12680942	*ASAP1*	0.84 [0.79–0.89]	–
Morocco	Pulmonary TB	556	650	rs358793	Intergenic	0.68 [0.57–0.82]	(77)
–	–	–	–	rs17590261	Intergenic	6.24 [2.38–16.33]	–
–	–	–	–	rs6786408	*FOXP1*	1.47 [1.23–1.79]	–
–	–	–	–	rs916943	*AGMO*	1.86 [1.33–2.6]	–
Uganda	HIV positive TB resistance	267	314	rs4921437	*IL12B*	0.37 [0.27–0.53]	(78)
Tanzania	–	–	–	–	–	–	–
Iceland	TST positivity	8162	277643	rs557011	Between *HLA-DQA1* and *HLA-DRB1*	1.25 [1.17–1.33]	(79)
–	–	–	–	rs9271378	Between *HLA-DQA1* and *HLA-DRB1*	0.78 [0.73–0.84]	–
–	–	–	–	rs9272785	*HLA-DQA1*	1.14 [1.09–1.19]	–
Uganda	TST reactivity	224	225	rs877356	*IL9*	0.27 [0.17–0.42]	(80)
Tanzania	–	–	–	–	–	–	–
23 and Me (European ancestry)	Positive TST	4426	84290	rs2894257	*HLA*	1.36 [1.33–1.39]	(81)
China (Han Chinese)	Pulmonary and extrapulmonary TB	4310	6386	rs4240897	*MFN2*	0.79 [0.75–0.83]	(82)
–	–	–	–	rs41553512	HLA class II	2.14 [1.78–2.57]	–
–	–	–	–	rs2269497	*RGS12*	1.51 [1.35–1.68]	–
Thailand	Non-Beijing lineage-infected old age onset	182	489	rs1418425	*CD53*	1.74 [1.43–2.12]	(83)

Compared to the study of *M. tuberculosis* infection resistance, a larger number of investigations have been published addressing the genetic factors that protect against or predispose to developing clinical TB. Indeed, 11 TB GWAS have been done using clinical TB as phenotype [Table 1, reviewed by (20, 21)]. Highlights included the identification of the 11p13 locus first identified in West Africa and replicated in Russia, Indonesia and South Africa (74, 75), a large Icelandic GWAS which identified HLA class II variants which was weakly replicated in Russia and Croatia (79) and a recent GWAS of TB resistance in HIV positive individuals from hyperendemic TB regions in Uganda and Tanzania (78). The latter study found an association with a locus at chromosome region 5q33.3. The associated variant is embedded in an H3K27A histone mark, but is also in a genomic region that includes *IL12B*, a gene known to underlie Mendelian susceptibility to mycobacterial disease (78).

Since the publication of previous reviews of clinical TB GWAS (20, 21), two additional studies have been completed using this study design. A three-stage replication approach was used in the Han Chinese and generated genotyping data (691 388 SNPs) for 972 TB cases and 1537 controls in the first stage (82). In the second stage, the top 45 loci were analyzed in 2278 TB cases and 2752 controls and the nine most significant variants were genotyped in 1060 TB cases and 2752 controls. Variants in three loci, namely *MFN2* (rs4240897, *p* = 1.41 × 10^−11^, OR 0.79, 95% CI 0.75–0.83), HLA class II (rs41553512, *p* = 7.93 × 10^−11^, OR 2.14, 95% CI 1.78–2.57), and *RGS12* (rs2269497, *p* = 3.37 × 10^−8^, OR 1.51, 95% CI 1.35–1.68) were significantly associated with TB in a meta-analysis of the three stages (4310 cases vs. 6386 controls). These are all excellent TB candidate genes and gene expression data supported the functional significance of two of the identified variants. The rs4240897 variant regulates *MFN2* expression suggesting that this variant could affect platelet count and macrophage differentiation. In addition, expression of this gene was increased in TB cases compared to controls (82). Another gene in close proximity to rs4240897 is *TNFRSF8* and expression of this immune gene was lower in TB cases than controls. Signaling of the *TNFSF8/TNFRSF8* pathway enhanced interferon gamma production in response to *M. bovis* BCG stimulation (82). A GWAS done in Thailand relied on *M. tuberculosis* pathogen lineage information and identified a chromosome 1p13 association between 489 healthy controls and 182 cases with non-Beijing lineage-infected old age onset (rs1418425, *p* = 2.54 × 10^−8^, OR 1.74, 95% CI 1.43–2.12) (83). The variant is located in the vicinity of the *CD53* gene and expression of this leukocyte surface glycoprotein was correlated with active TB (83). In addition, the rs1418425 variant is a known cis-expression quantitative trait locus in *M. tuberculosis* infected dendritic cells (83).

Clearly there is very little overlap with respect to the loci detected between the individual GWAS, but it seems that replication is more likely when populations with similar genetic backgrounds are compared. This was seen for the *WT1* locus in West and South Africa and it is possible that the same HLA class II factors are being tagged in Icelandic and other European populations, but this is not known at this point (74, 75, 79, 81). When GWAS data from Han Chinese and Gambians were combined in a meta-analysis, no significant associations were detected (82). Deciphering the complete genetic architecture of a complex trait requires more than a single ancestry, as was the case for skin pigmentation genes and other phenotypes (90, 91). For this reason, population genetics also has to be considered in investigations of TB resistance (92). Excess European, South Asian and East Asian ancestry protects admixed South African individuals against active TB, whereas excess African ancestry increased the risk for developing disease. These disparities in disease incidence were harnessed in a TB admixture mapping study (75, 93). The contribution of ethnicity to TB resistance may be due to selection after centuries of exposure to *M. tuberculosis* (as discussed in the section “Early epidemiological evidence of natural TB resistance”). This is supported by findings that individuals with diverse genetic backgrounds have different rates of TB infection and disease progression (not affected by socio economic circumstances) and the intensity of immune responses differ (35, 52–54, 59).

## Functional genetics

The mechanisms through which genetic variation contributes to TB resistance require functional follow-up to support statistical findings of epidemiological studies. Investigations of epigenetics, microRNAs, and other products of transcription can provide functions to these genetic variants, but can also identify novel genes and pathways involved with TB resistance (94).

### Transcriptomics

Transcriptional profiles generated from blood cells have contributed to the elucidation of pathways involved in resistance to infection. Genome-wide transcriptional profiles from infected monocytes isolated from TST positive and persistently negative household contacts from Uganda who did not develop TB at least two years after follow-up were generated using microarrays (86). Pathways controlled by histone deacetylase were associated with resistance to *M. tuberculosis* infection and indicated that this function is vital in the early innate immune response to infection (86). Although this anti-inflammatory mechanism holds promise as a therapy, the *in vitro* findings may not extend to effects *in vivo*. The use of histone deacetylase inhibitors did not increase survival in a sepsis model (95) and histone deacetylase-related genes were also expressed in TST positive individuals (86). In a non-human primate model, a signature of 34 pre-infection transcripts could differentiate between animals that would progress to active disease or develop LTBI (96). Twelve of the upregulated transcripts were associated with interferon, cell cycle and inflammation processes. When the outcome was stratified based on ^18^F-fluorodeoxyglucose (FDG) positron emission tomography coupled with computed tomography (PET CT), 30 pre-infection transcripts were differentially expressed between animals with low and high FDG avidity. The differentially expressed genes did not correspond to the clinical status or lung avidity groups, but function in the same pathways related to inflammation and interferon (96). The inherent genetic ability of the host to upregulate these pathways may correspond to poor infection outcomes (96). Importantly, both studies underline that a balanced inflammatory response, regulated by the host genome, is critical to determine the outcome of infection (86, 96).

Although genome-wide transcriptomic studies of TB infection resistance are in their infancy, many studies have identified blood gene expression signatures for the classification of the TB pathogenesis stages (including the diagnosis of active TB) and monitoring of treatment efficacy (97–112). The value of these studies lies in their predictive accuracy, since TST and IGRAs cannot fulfill this function (21). However, transcriptomics cannot detect those genetically determined for disease progression before the onset of the process (21). Several predictive signatures of TB risk have been developed from whole blood RNA sequencing. Recently a four-transcript signature, labeled RISK4, could predict disease progression up to 2 years before TB symptoms presented in Africa cohorts from South Africa, The Gambia and Ethiopia (113). This signature consists of two upregulated (growth arrest–specific 6 and septin 4) and two downregulated (cluster of differentiation 1C and B lymphocyte kinase) genes. A 16 gene predictive signature of TB risk was developed from whole blood RNA sequencing of adolescents and could not only identify individuals at risk of developing active TB after LTBI, but could also distinguish active disease from LTBI and other disease forms in two African populations and three validation samples (114). The sixteen genes were Ankyrin repeat domain 22, Apolipoprotein L1, basic leucine zipper ATF-like transcription factor 2, ETS Variant 7, Fc Fragment Of IgG Receptor Ia, Fc Fragment Of IgG Receptor Ib, Guanylate Binding Protein 1,2,4, and 5, Scavenger Receptor Class F Member 1, septin 4, Serpin Family G Member 1, Signal Transducer And Activator Of Transcription 1, Transporter 1, ATP Binding Cassette Subfamily B Member and TRAF-Type Zinc Finger Domain Containing 1. In HIV positive drug users with and without TB, expression of the IL-13 and autoimmune regulator genes were predictive of developing disease even 8 months before the actual diagnosis (115), but this signature was not validated in the RNA sequencing study of HIV negative adolescents (114). To identify correlates of TB resistance using the LTBI phenotype as a proxy, network analysis was applied to a number of TB transcriptomic datasets. Here the focus was specifically on gene expression profiles of macrophages, as these cells can mount an antimicrobial response (116). IL-32 was identified as a functional marker of resistance to active TB and mediated interferon gamma vitamin D dependent antimicrobial immunity (16, 116). An *in vitro* investigation of monocytes (isolated from LTBI and active TB individuals) after *M. tuberculosis* infection indicated that IL-26 downregulation was beneficial to anti-mycobacterial activity, making it a plausible susceptibility candidate gene (94). A meta-analysis of 16 published studies identified a set of 380 genes that were differentially expressed in active TB in most investigations with interferon gamma as the most significant potential upstream regulated molecule (117).

### Epigenetics

The contribution of epigenetic mechanisms to the regulation of inflammatory immune responses in TB is an emerging field and evaluations of the genetic regulation of transcriptomic responses can assist in revealing the biology of TB host resistance (118). Epigenetic regulation incorporates all chromosomal modifications that alter gene expression without changing the underlying coding DNA nucleotide sequence, such as DNA methylation and histone acetylation. Methylation events in monocytes and granulocytes could discriminate between TB cases and healthy LTBI controls (119).

Histone modifications have been linked to mTOR dependent regulation of glucose and glutamine metabolism in BCG-trained monocytes and macrophages, with histone H3 trimethylation of lysine 4 (H3K4me3) found to be significantly increased at the promotors of mTOR, HK2 and PFKP, while trimethylation of lysine 9 (H3Kme3) was significantly decreased (120). Histone H3 hypoacetylation, specifically at lysine 14 (H3K14ac) was associated with active pulmonary TB (121) as well as being essential for the activation of several pro-inflammatory cytokines (122, 123). Interestingly, the macrophage response to different immune challenges can result in the generation of histone marks associated with *de novo* enhancer elements (124, 125). These marks have been hypothesized to cause the epigenetic reprogramming of the macrophages leading to a stronger transcriptional response to a second stimulus (124). In addition to histone modifications, recent data suggests that DNA methylation also plays a significant role in in the reprogramming of innate immune cells and the regulation of transcriptional programs following *M. tuberculosis* infection (126).

Epigenetic modification of histone acetylation in monocyte-derived macrophages plays a significant regulatory role in *M. tuberculosis*-dependent gene expression and in the secretion of matrix metalloproteinase enzymes driving immunopathology. Modification of histone acetylases has implications for TB resistance too, based on the findings of Seshadri et al. discussed above (86). Variants in the regulatory regions of over 700 genes that were up- or downregulated after *M. tuberculosis* infection of monocyte-derived dendritic cells significantly influenced gene expression regardless of the stimulation status of cells. (127). These variants are expression quantitative trait loci (eQTL) and a subset of these (response-eQTL) were dependent on *M. tuberculosis* stimulation, indicating that epigenetic effects contribute to TB pathogenesis (126). Manipulating these regulatory mechanisms may have potential as host-directed therapy (128).

### MicroRNAs

MicroRNAs (miRNAs) play a crucial role in TB pathogenesis (129). MiRNAs are short, non-coding RNA molecules that regulate mRNA translation and degradation and affect the function of many immune cell types (129). Used as markers, miRNAs can distinguish between active disease, LTBI or other microbial infections (130–137), and also influence TB susceptibility, specifically miR-155 and miR-223 (138–141). MiR-223 directly targeted chemoattractants such as CXCL2, CCL3, and IL-6 to control neutrophil driven inflammation (138, 139). When miR-223 was deleted in a TB resistant mouse model these animals became extremely susceptible to TB, but the phenotype could be partly restored through the neutralization of the abovementioned chemoattractants (138). miR-155 was highly expressed after mycobacterial infection both *in vivo* and *in vitro* (139). The induced expression of miR-155 enhanced the autophagic response in macrophages thereby stimulating mycobacterial phagosome maturation and reducing the survival rate of intracellular mycobacteria. In contrast, when miR-155 was inhibited, there was increased mycobacterial survival. The mechanism of action of miR-155 is through the targeting of Ras homolog enrich in brain (Rheb), a known negative regulator of autophagy. When miR-155 was bound to the 3'-untranslated region of Rheb, both autophagy and intracellular killing of mycobacteria were increased (139). In addition to expression analysis, genetic variants in miR-499 and miR-146a were associated with pulmonary TB susceptibility in a case-control association study (142).

### Transgenic animals

Although the animal models (discussed in the section “Early epidemiological evidence of natural TB resistance”) were initially used to study the natural occurrence of TB resistance, advances in molecular genetic techniques have facilitated the creation of resistant species. Transgenic cattle with a knock-in SP110 nuclear body protein (*SP110*) gene were created using transcription activator-like effector nuclease (TALEN)-mediated genome modification (143). The mouse homolog of this gene, Ipr1, was previously shown to mediate innate immunity in sst1 congenic mice and *SP110* variants were associated in some settings with human pulmonary TB (144, 145), but not others (146–149). In the transgenic cattle apoptosis instead of necrosis was activated after infection (143). *In vivo* and *in vitro* experiments indicated that these animals could control growth and proliferation of *M. bovis*. Significantly, transmission experiments using tuberculous cattle indicated that the transgenic animals were resistant to low dose *M. bovis* infection (143).

## Controversies, current research gaps, and future developments

The four complementary theories of infectious disease propose that inter-individual variability in presentation depends on four factors, namely microbiological, ecological, immunological, and genetic (150). These elements not only intersect but are all required to dissect a complex infection phenotype such as TB resistance (150). To delineate the contribution of genetic factors will require innovative new approaches to combine available data sets to understand the resistance phenomenon, in particular in HIV-infected persons. Integrated clinical and laboratory defined phenotypes, whole genome sequencing, epigenetic and transcriptomic studies will be required to address this challenge.

The co-evolution of *M. tuberculosis* and humans has shaped host-pathogen interactions for thousands of years and has likely contributed to the diverse range of responses after infection including the phenotype of the TB resister (50, 61, 151). Host-pathogen interaction investigations are however complicated by the genetic heterogeneity of the bacterium (50). One approach is to use *M. tuberculosis* pathogen lineage information as was done for a TB GWAS in Thailand (Table 1) and several candidate gene association studies (83, 152–155). Despite the challenges, developments in this area could in future be used to design targeted vaccines and therapies directed to specific populations or individuals (50).

Several TB GWAS have interrogated resistance to disease, but most of these have been underpowered due to the extreme phenotypic heterogeneity. Meta-analyses can provide a solution to this problem and could provide insight into population-specific associations by harnessing linkage disequilibrium to fine map associations. The International TB Host Genetics Consortium has been established to collate TB GWAS data from individuals with pulmonary TB and healthy controls to do a large-scale meta-analysis (156). This large-scale approach will not be feasible to investigate persistently TST/IGRA negative individuals, since phenotyping is a costly process and requires several repeat assays to exclude those who revert and convert. Fortunately, since the TB resister phenotype is at the end of the TB susceptibility spectrum, it is possible that variants contributing to this extreme phenotype can be detected in limited sample sizes, as has been seen in investigations of HIV-infected participants who do not become infected or progress to active TB despite living in a TB endemic region (86, 78).

Finally, genome sequencing technologies, which are already used to diagnose individuals with Mendelian susceptibility to mycobacterial diseases, will deliver resistance variants not captured by microarray genotyping and imputing. Once genomes from TB resisters are available for data mining and analysis in system biology approaches—which will include transcriptomics, epigenomics, microbiomics, and other omics, we may be able to achieve prediction of individuals genetically determined as resistant.

## Conclusion

The involvement of a human genetic component in susceptibility to infection with *M. tuberculosis* and progression to active disease is incontestable. Findings from clinical genetics, genetic epidemiology, population and functional genetics have all contributed to identify TB susceptibility genes. More intriguing is the other side of the phenotypic coin—that of resistance to either initial infection or, after infection, resistance to progression to disease. Although the phenomenon is now recognized, the exact genetic variants and mechanisms that contribute still require elucidation. The most successful approaches in resistance/susceptibility investigation have focused on specific infection and disease phenotypes and the resister phenotype may hold the key to the discovery of actionable genetic variants in TB infection and disease.

## Author contributions

All authors listed have made a substantial, direct and intellectual contribution to the work, and approved it for publication.

### Conflict of interest statement

The authors declare that the research was conducted in the absence of any commercial or financial relationships that could be construed as a potential conflict of interest. The reviewer CM declared a past co-authorship with the authors MM, PvH and EH to the handling editor.

## References

[1] WHO Global Tuberculosis Report 2017. Available online at: http://www.who.int/tb/publications/global_report/en/ (Accessed November 7, 2017).

[2] Mathema B Kurepina NE Bifani PJ Kreiswirth BN. Molecular epidemiology of tuberculosis: current insights. Clin Microbiol Rev. (2006) 19:658–85. 10.1128/CMR.00061-05 17041139 PMC1592690

[3] Pai M Behr MA Dowdy D Dheda K Divangahi M Boehme CC Tuberculosis. Nat Rev Dis Primers (2016) 2:16076 10.1038/nrdp.2016.76 27784885

[4] Möller M de Wit E Hoal EG. Past, present and future directions in human genetic susceptibility to tuberculosis. FEMS Immunol Med Microbiol. (2010) 58:3–26. 10.1111/j.1574-695X.2009.00600.x 19780822

[5] van Helden PD Möller M Babb C Warren R Walzl G Uys P. TB epidemiology and human genetics. In Chadwick DJ Goode J, editors. Innate Immunity to Pulmonary Infection. Chichester: Wiley (2006), 17–41. 17278383

[6] Smith I. *Mycobacterium tuberculosis* pathogenesis and molecular determinants of virulence. Clin Microbiol Rev. (2003) 16:463–96. 10.1128/CMR.16.3.463-496.2003 12857778 PMC164219

[7] Frieden TR Sterling TR Munsiff SS Watt CJ Dye C. Tuberculosis. Lancet (2003) 362:887–99. 10.1016/S0140-6736(03)14333-4 13678977

[8] Houben RMGJ Dodd PJ. The global burden of latent tuberculosis infection: a re-estimation using mathematical modelling. PLoS Med. (2016) 13:e1002152. 10.1371/journal.pmed.1002152 27780211 PMC5079585

[9] Theron G Peter J van Zyl-Smit R Mishra H Streicher E Murray S. Evaluation of the Xpert MTB/RIF assay for the diagnosis of pulmonary tuberculosis in a high HIV prevalence setting. Am J Respir Crit Care Med. (2011) 184:132–40. 10.1164/rccm.201101-0056OC 21493734

[10] Nayak S Acharjya B. Mantoux test and its interpretation. Indian Dermatol Online J. (2012) 3:2–6. 10.4103/2229-5178.93479 23130251 PMC3481914

[11] Cadena AM Fortune SM Flynn JL. Heterogeneity in tuberculosis. Nat Rev Immunol. (2017) 17:691–702. 10.1038/nri.2017.69 28736436 PMC6247113

[12] Esmail H Barry CE Young DB Wilkinson RJ. The ongoing challenge of latent tuberculosis. Philos Trans R Soc Lond B Biol Sci. (2014) 369:20130437. 10.1098/rstb.2013.0437 24821923 PMC4024230

[13] Esmail H Barry CE Wilkinson RJ. Understanding latent tuberculosis: the key to improved diagnostic and novel treatment strategies. Drug Discov Today (2012) 17:514–21. 10.1016/j.drudis.2011.12.013 22198298 PMC3443737

[14] Barry CE Boshoff H Dartois V Dick T Ehrt S Flynn J The spectrum of latent tuberculosis: rethinking the goals of prophylaxis. Nat Rev Microbiol. (2009) 7:845–55. 10.1038/nrmicro2236 19855401 PMC4144869

[15] Young DB Gideon HP Wilkinson RJ. Eliminating latent tuberculosis. Trends Microbiol. (2009) 17:183–8. 10.1016/j.tim.2009.02.005 19375916

[16] Deffur A Wilkinson RJ Coussens AK. Tricks to translating TB transcriptomics. Ann Transl Med. (2015) 3(Suppl. 1):S43. 10.3978/j.issn.2305-5839.2015.04.12 26046091 PMC4437947

[17] Simmons JD Stein CM Seshadri C Campo M Alter G Fortune S Immunological mechanisms of human resistance to persistent *Mycobacterium tuberculosis* infection. Nat Rev Immunol. (2018) 18 575–89. 10.1038/s41577-018-0025-3 PMC6278832 29895826

[18] Cadena AM Flynn JL Fortune SM. The importance of first impressions: early events in *Mycobacterium tuberculosis* infection influence outcome. MBio (2016) 7:e00342-16. 10.1128/mBio.00342-16 27048801 PMC4817258

[19] Abel L El-Baghdadi J Bousfiha AA Casanova J-L Schurr E. Human genetics of tuberculosis: a long and winding road. Philos Trans R Soc Lond B Biol Sci. (2014) 369:20130428. 10.1098/rstb.2013.0428 24821915 PMC4024222

[20] Kinnear C Hoal EG Schurz H van Helden PD Möller M. The role of human host genetics in tuberculosis resistance. Expert Rev Respir Med. (2017) 11:721–37. 10.1080/17476348.2017.1354700 28703045

[21] Orlova M Schurr E. Human genomics of *Mycobacterium tuberculosis* infection and disease. Curr Genet Med Rep. (2017) 5:125–31. 10.1007/s40142-017-0124-7 29201558 PMC5703416

[22] Morrison J Pai M Hopewell PC. Tuberculosis and latent tuberculosis infection in close contacts of people with pulmonary tuberculosis in low-income and middle-income countries: a systematic review and meta-analysis. Lancet Infect Dis. (2008) 8:359–68. 10.1016/S1473-3099(08)70071-9 18450516

[23] Lienhardt C Fielding K Sillah JS Bah B Gustafson P Warndorff D. Investigation of the risk factors for tuberculosis: a case-control study in three countries in West Africa. Int J Epidemiol. (2005) 34:914–23. 10.1093/ije/dyi100 15914505

[24] Hill PC Brookes RH Fox A Fielding K Jeffries DJ Jackson-Sillah D. Large-scale evaluation of enzyme-linked immunospot assay and skin test for diagnosis of *Mycobacterium tuberculosis* infection against a gradient of exposure in The Gambia. Clin Infect Dis. (2004) 38:966–73. 10.1086/382362 15034828

[25] Aziz A Ishaq M Akhwand R. Infection risk of sputum positive tuberculosis patients to their family contacts with and without chemotherapy. J Pak Med Assoc. (1985) 35:249–52. 3932702

[26] Devadatta S Dawson JJY Fox W Janardhanam B Radhakrishna S Ramakrishnan CV Attack rate of tuberculosis in a 5-year period among close family contacts of tuberculous patients under domiciliary treatment with isoniazid plus PAS or isoniazid alone. Bull World Health Organ. (1970) 42:337–51. 5310206 PMC2427525

[27] Verrall AJ Netea MG Alisjahbana B Hill PC van Crevel R. Early clearance of *Mycobacterium tuberculosis*: a new frontier in prevention. Immunology (2014) 141:506–13. 10.1111/imm.12223 24754048 PMC3956425

[28] Hanifa Y Grant AD Lewis J Corbett EL Fielding K Churchyard G. Prevalence of latent tuberculosis infection among gold miners in South Africa. Int J Tuberc Lung Dis. (2009) 13:39–46. 19105877

[29] Houk VN Baker JH Sorensen K Kent DC. The epidemiology of tuberculosis infection in a closed environment. Arch Environ Health (1968) 16:26–35. 5638222 10.1080/00039896.1968.10665011

[30] DiStasio AJ Trump DH. The investigation of a tuberculosis outbreak in the closed environment of a U.S. Navy ship, 1987. Mil Med. (1990) 155:347–51. 2119013

[31] Dickie HA. Tuberculosis in student nurses and medical students at the University of Wisconsin. Ann Intern Med. (1950) 33:941–59. 10.7326/0003-4819-33-4-941 14771762

[32] Myers JA The prevention of tuberculosis among nurses. Am J Nurs. (1930) 30:1361–72.

[33] Heimbeck J Incidence of tuberculosis in young adult women, with special reference to employment. Br J Tuber. (1938) 32:154166 10.1016/S0366-0850(38)80144-7

[34] Badger TL Spink WW First-infection type of tuberculosis in adults. N Engl J Med. (1937) 217:424–31. 10.1056/NEJM193709092171102

[35] Dubos R Dubos J The White Plague: Tuberculosis, Man and Society. Boston, MA: Little, Brown & Co (1952).

[36] Kallmann FJ Reisner D Twin studies on the significance of genetic factors in tuberculosis. Am Rev Tuberc. (1943) 47:549–47.

[37] Murray CJ Styblo K Rouillon A. Tuberculosis in developing countries: burden, intervention and cost. Bull Int Union Tuber Lung Dis. (1990) 65:6–24. 2190653

[38] Chackerian AA Behar SM. Susceptibility to *Mycobacterium tuberculosis*: lessons from inbred strains of mice. Tuberculosis (2003) 83:279–85. 10.1016/S1472-9792(03)00017-9 12972341

[39] Lurie MB Abramson S Heppleston AG. On the response of genetically resistant and susceptible rabbits to the quantitative inhalation of human type tubercle bacilli and the nature of resistance to tuberculosis. J Exp Med. (1952) 95:119–34. 10.1084/jem.95.2.119 14907965 PMC2212059

[40] Lurie MB Heredity, constitution and tuberculosis. an experiments study. Am Rev Tuber. (Suppl) (1941) 44:125.

[41] Lurie MB. On the mechanism of immunity in tuberculosis: the host-parasite relationship under the conditions of a localized agar focus of infection and the generalization of the disease in normal and immunized rabbits. J Exp Med. (1936) 63:923–46. 19870514 10.1084/jem.63.6.923 PMC2133408

[42] Lurie MB. Experimental epidemiology of tuberculosis: hereditary resistance to attack by tuberculosis and to the ensuing disease and the effect of the concentration of tubercle bacilli upon these two phases of resistance. J Exp Med. (1944) 79:573–89. 19871388 10.1084/jem.79.6.573 PMC2135381

[43] Werneck-Barroso E. Innate resistance to tuberculosis: revisiting Max Lurie genetic experiments in rabbits. Int J Tuberc Lung Dis. (1999) 3:166–8. 10091885

[44] Fox GJ Orlova M Schurr E. Tuberculosis in newborns: the lessons of the “Lübeck Disaster” (1929-1933). PLoS Pathog. (2016) 12:e1005271. 10.1371/journal.ppat.1005271 26794678 PMC4721647

[45] Motulsky AG. Metabolic polymorphisms and the role of infectious diseases in human evolution. Hum Biol. (1960) 32:28–62. 14424690

[46] Stead WW. The origin and erratic global spread of tuberculosis. How the past explains the present and is the key to the future. Clin Chest Med. (1997) 18:65–77. 9098611 10.1016/s0272-5231(05)70356-7

[47] Sousa AO Salem JI Lee FK Verçosa MC Cruaud P Bloom BR. An epidemic of tuberculosis with a high rate of tuberculin anergy among a population previously unexposed to tuberculosis, the Yanomami Indians of the Brazilian Amazon. Proc Natl Acad Sci USA. (1997) 94:13227–32. 9371828 10.1073/pnas.94.24.13227 PMC24291

[48] Comas I Hailu E Kiros T Bekele S Mekonnen W Gumi B. Population genomics of *Mycobacterium tuberculosis* in Ethiopia contradicts the virgin soil hypothesis for human tuberculosis in Sub-Saharan Africa. Curr Biol. (2015) 25:3260–6. 10.1016/j.cub.2015.10.061 26687624 PMC4691238

[49] Jaeger LH de Souza SMFM Dias OF Iñiguez AM. *Mycobacterium tuberculosis* complex in remains of 18th-19th century slaves, Brazil. Emerg Infect Dis. (2013) 19:837–9. 10.3201/eid1905.120193 23697340 PMC3647487

[50] Hoal EG Dippenaar A Kinnear C van Helden PD Möller M. The arms race between man and *Mycobacterium tuberculosis*: time to regroup. Infect Genet Evol. (2017). 10.1016/j.meegid.2017.08.021. [Epub ahead of print]. 28843547

[51] Hurtado A Magdalena, Hill Kim R., Rosenblatt W, Bender J, Scharmen T. Longitudinal study of tuberculosis outcomes among immunologically naive Aché natives of Paraguay. Am J Phys Anthropol. (2003) 121:134–50. 10.1002/ajpa.10228 12740957

[52] Keppel KG. Ten largest racial and ethnic health disparities in the United States based on Healthy People 2010 Objectives. Am J Epidemiol. (2007) 166:97–103. 10.1093/aje/kwm044 17463050

[53] Stead WW Senner JW Reddick WT Lofgren JP. Racial differences in susceptibility to infection by *Mycobacterium tuberculosis*. N Engl J Med. (1990) 322:422–7. 10.1056/NEJM199002153220702 2300105

[54] Serpa JA Teeter LD Musser JM Graviss EA. Tuberculosis disparity between US-born blacks and whites, Houston, Texas, USA. Emerg Infect Dis. (2009) 15:899–904. 10.3201/eid1506.081617 19523288 PMC2727328

[55] Uren C Möller M van Helden PD Henn BM Hoal EG. Population structure and infectious disease risk in southern Africa. Mol Genet Genomics (2017) 292:499–509. 10.1007/s00438-017-1296-2 28229227

[56] Lipsitch M Sousa AO. Historical intensity of natural selection for resistance to tuberculosis. Genetics (2002) 161:1599–607. 12196403 10.1093/genetics/161.4.1599 PMC1462208

[57] Barreiro LB Quintana-Murci L. From evolutionary genetics to human immunology: how selection shapes host defence genes. Nat Rev Genet. (2010) 11:17–30. 10.1038/nrg2698 19953080

[58] Manry J Laval G Patin E Fornarino S Itan Y Fumagalli M. Evolutionary genetic dissection of human interferons. J Exp Med. (2011) 208:2747–59. 10.1084/jem.20111680 22162829 PMC3244034

[59] Nédélec Y Sanz J Baharian G Szpiech ZA Pacis A Dumaine A. Genetic ancestry and natural selection drive population differences in immune responses to pathogens. Cell (2016) 167:657–69.e21. 10.1016/j.cell.2016.09.025 27768889

[60] Deschamps M Laval G Fagny M Itan Y Abel L Casanova J-L. Genomic signatures of selective pressures and introgression from archaic hominins at human innate immunity genes. Am J Hum Genet. (2016) 98:5–21. 10.1016/j.ajhg.2015.11.014 26748513 PMC4716683

[61] Nahid P Jarlsberg LG Kato-Maeda M Segal MR Osmond DH Gagneux S. Interplay of strain and race/ethnicity in the innate immune response to *M. tuberculosis*. PLoS ONE (2018) 13:e0195392. 10.1371/journal.pone.0195392 29787561 PMC5963792

[62] Sepulveda RL Heiba IM Navarrete C Elston RC Gonzalez B Sorensen RU. Tuberculin reactivity after newborn BCG immunization in mono- and dizygotic twins. Tuber Lung Dis. (1994) 75:138–43. 10.1016/0962-8479(94)90043-4 8032047

[63] Jepson A Fowler A Banya W Singh M Bennett S Whittle H. Genetic regulation of acquired immune responses to antigens of *Mycobacterium tuberculosis*: a study of twins in West Africa. Infect Immun. (2001) 69:3989–94. 10.1128/IAI.69.6.3989-3994.2001 11349068 PMC98461

[64] Cobat A Barrera LF Henao H Arbeláez P Abel L García LF. Tuberculin skin test reactivity is dependent on host genetic background in Colombian tuberculosis household contacts. Clin Infect Dis. (2012) 54:968–71. 10.1093/cid/cir972 22291100 PMC3297651

[65] Cobat A Gallant CJ Simkin L Black GF Stanley K Hughes J High heritability of anti-mycobacterial immunity in a hyper-endemic area for tuberculosis disease. J Infect Dis. (2010) 201:15–9. 10.1086/648611 19938975

[66] Tao L Zalwango S Chervenak K Thiel B Malone LL Qiu F. Genetic and shared environmental influences on interferon-γ production in response to *Mycobacterium tuberculosis* antigens in a Ugandan population. Am J Trop Med Hyg. (2013) 89:169–73. 10.4269/ajtmh.12-0670 23629934 PMC3748477

[67] Zembrzuski VM Basta PC Callegari-Jacques SM Santos RV Coimbra CEA Salzano FM. Cytokine genes are associated with tuberculin skin test response in a native Brazilian population. Tuberculosis (2010) 90:44–9. 10.1016/j.tube.2009.11.002 20005781

[68] Thye T Browne EN Chinbuah MA Gyapong J Osei I Owusu-Dabo E IL10 haplotype associated with tuberculin skin test response but not with pulmonary TB. PLoS ONE (2009) 4:e5420 10.1371/journal.pone.0005420 19412539 PMC2671601

[69] Horne DJ Graustein AD Shah JA Peterson G Savlov M Steele S. Human ULK1 Variation and Susceptibility to *Mycobacterium tuberculosis* Infection. J Infect Dis. (2016) 214:1260–7. 10.1093/infdis/jiw347 27485354 PMC5034956

[70] Thye T Vannberg FO Wong SH Owusu-Dabo E Osei I Gyapong J. Genome-wide association analyses identifies a susceptibility locus for tuberculosis on chromosome 18q11.2. Nat Genet. (2010) 42:739–41. 10.1038/ng.639 20694014 PMC4975513

[71] Oki NO Motsinger-Reif AA Antas PR Levy S Holland SM Sterling TR. Novel human genetic variants associated with extrapulmonary tuberculosis: a pilot genome wide association study. BMC Res Notes (2011) 4:28. 10.1186/1756-0500-4-28 21281516 PMC3041678

[72] Mahasirimongkol S Yanai H Mushiroda T Promphittayarat W Wattanapokayakit S Phromjai J. Genome-wide association studies of tuberculosis in Asians identify distinct at-risk locus for young tuberculosis. J Hum Genet. (2012) 57:363–7. 10.1038/jhg.2012.35 22551897

[73] Png E Alisjahbana B Sahiratmadja E Marzuki S Nelwan R Balabanova Y. A genome wide association study of pulmonary tuberculosis susceptibility in Indonesians. BMC Med Genet. (2012) 13:5. 10.1186/1471-2350-13-5 22239941 PMC3287960

[74] Thye T Owusu-Dabo E Vannberg FO van Crevel R Curtis J Sahiratmadja E. Common variants at 11p13 are associated with susceptibility to tuberculosis. Nat Genet. (2012) 44:257–9. 10.1038/ng.1080 22306650 PMC3427019

[75] Chimusa ER Zaitlen N Daya M Möller M van Helden PD Mulder NJ. Genome-wide association study of ancestry-specific TB risk in the South African Coloured population. Hum Mol Genet. (2014) 23:796–809. 10.1093/hmg/ddt462 24057671 PMC3888262

[76] Curtis J Luo Y Zenner HL Cuchet-Lourenço D Wu C Lo K. Susceptibility to tuberculosis is associated with variants in the *ASAP1* gene encoding a regulator of dendritic cell migration. Nat Genet. (2015) 47:523–7. 10.1038/ng.3248 25774636 PMC4414475

[77] Grant AV Sabri A Abid A Abderrahmani Rhorfi I Benkirane M Souhi H. A genome-wide association study of pulmonary tuberculosis in Morocco. Hum Genet. (2016) 135:299–307. 10.1007/s00439-016-1633-2 26767831 PMC5042142

[78] Sobota RS Stein CM Kodaman N Scheinfeldt LB Maro I Wieland-Alter W. A Locus at 5q33.3 confers resistance to tuberculosis in highly susceptible individuals. Am J Hum Genet. (2016) 98:514–24. 10.1016/j.ajhg.2016.01.015 26942285 PMC4800052

[79] Sveinbjornsson G Gudbjartsson DF Halldorsson BV Kristinsson KG Gottfredsson M Barrett JC. HLA class II sequence variants influence tuberculosis risk in populations of European ancestry. Nat Genet. (2016) 48:318–22. 10.1038/ng.3498 26829749 PMC5081101

[80] Sobota RS Stein CM Kodaman N Maro I Wieland-Alter W Igo RP. A chromosome 5q31.1 locus associates with tuberculin skin test reactivity in HIV-positive individuals from tuberculosis hyper-endemic regions in east Africa. PLoS Genet. (2017) 13:e1006710. 10.1371/journal.pgen.1006710 28628665 PMC5495514

[81] Tian C Hromatka BS Kiefer AK Eriksson N Noble SM Tung JY. Genome-wide association and HLA region fine-mapping studies identify susceptibility loci for multiple common infections. Nat Commun. (2017) 8:599. 10.1038/s41467-017-00257-5 28928442 PMC5605711

[82] Qi H Zhang Y-B Sun L Chen C Xu B Xu F. Discovery of susceptibility loci associated with tuberculosis in Han Chinese. Hum Mol Genet. (2017) 26:4752–63. 10.1093/hmg/ddx365 29036319

[83] Omae Y Toyo-Oka L Yanai H Nedsuwan S Wattanapokayakit S Satproedprai N. Pathogen lineage-based genome-wide association study identified CD53 as susceptible locus in tuberculosis. J Hum Genet. (2017) 62:1015–22. 10.1038/jhg.2017.82 28878339 PMC5709719

[84] Stein CM Zalwango S Malone LL Won S Mayanja-Kizza H Mugerwa RD. Genome scan of *M. tuberculosis* infection and disease in Ugandans. PLoS ONE (2008) 3:e4094. 10.1371/journal.pone.0004094 19116662 PMC2605555

[85] Igo RP Hall NB Malone LL Hall JB Truitt B Qiu F. Fine-mapping analysis of a chromosome 2 region linked to resistance to *Mycobacterium tuberculosis* infection in Uganda reveals potential regulatory variants. Genes Immun. (2018) 10.1038/s41435-018-0040-1. [Epub ahead of print]. 30100616 PMC6374218

[86] Seshadri C Sedaghat N Campo M Peterson G Wells RD Olson GS. Transcriptional networks are associated with resistance to *Mycobacterium tuberculosis* infection. PLoS ONE (2017) 12:e0175844. 10.1371/journal.pone.0175844 28414762 PMC5393882

[87] Cobat A Gallant CJ Simkin L Black GF Stanley K Hughes J. Two loci control tuberculin skin test reactivity in an area hyperendemic for tuberculosis. J Exp Med. (2009) 206:2583–91. 10.1084/jem.20090892 19901083 PMC2806605

[88] Cobat A Poirier C Hoal E Boland-Auge A de La Rocque F Corrard F. Tuberculin skin test negativity is under tight genetic control of chromosomal region 11p14-15 in settings with different tuberculosis endemicities. J Infect Dis. (2015) 211:317–21. 10.1093/infdis/jiu446 25143445 PMC4279780

[89] Cobat A Hoal EG Gallant CJ Simkin L Black GF Stanley K. Identification of a major locus, TNF1, that controls BCG-triggered tumor necrosis factor production by leukocytes in an area hyperendemic for tuberculosis. Clin Infect Dis. (2013) 57:963–70. 10.1093/cid/cit438 23800941 PMC3765013

[90] Crawford NG Kelly DE Hansen MEB Beltrame MH Fan S Bowman SL. Loci associated with skin pigmentation identified in African populations. Science (2017) 358:eaan8433. 10.1126/science.aan8433 29025994 PMC5759959

[91] Martin AR Lin M Granka JM Myrick JW Liu X Sockell A. An unexpectedly complex architecture for skin pigmentation in Africans. Cell (2017) 171:1340–1353.e14. 10.1016/j.cell.2017.11.015 29195075 PMC5884124

[92] Daya M van der Merwe L van Helden PD Möller M Hoal EG. The role of ancestry in TB susceptibility of an admixed South African population. Tuberculosis (2014) 94:413–20. 10.1016/j.tube.2014.03.012 24832562

[93] Daya M van der Merwe L Gignoux CR van Helden PD Möller M Hoal EG. Using multi-way admixture mapping to elucidate TB susceptibility in the South African Coloured population. BMC Genomics (2014) 15:1021. 10.1186/1471-2164-15-1021 25422094 PMC4256931

[94] Guerra-Laso JM Raposo-García S García-García S Diez-Tascón C Rivero-Lezcano OM. Microarray analysis of *Mycobacterium tuberculosis*-infected monocytes reveals *IL26* as a new candidate gene for tuberculosis susceptibility. Immunology (2015) 144:291–301. 10.1111/imm.12371 25157980 PMC4298423

[95] Roger T Lugrin J Le Roy D Goy G Mombelli M Koessler T. Histone deacetylase inhibitors impair innate immune responses to Toll-like receptor agonists and to infection. Blood (2011) 117:1205–17. 10.1182/blood-2010-05-284711 20956800

[96] Gideon HP Skinner JA Baldwin N Flynn JL Lin PL. Early whole blood transcriptional signatures are associated with severity of lung inflammation in *Cynomolgus macaques* with *Mycobacterium tuberculosis* infection. J Immunol. (2016) 197:4817–28. 10.4049/jimmunol.1601138 27837110 PMC5289749

[97] Mistry R Cliff JM Clayton CL Beyers N Mohamed YS Wilson PA. Gene-expression patterns in whole blood identify subjects at risk for recurrent tuberculosis. J Infect Dis. (2007) 195:357–65. 10.1086/510397 17205474

[98] Kim M-J Wainwright HC Locketz M Bekker L-G Walther GB Dittrich C. Caseation of human tuberculosis granulomas correlates with elevated host lipid metabolism. EMBO Mol Med. (2010) 2:258–74. 10.1002/emmm.201000079 20597103 PMC2913288

[99] Berry MPR Graham CM McNab FW Xu Z Bloch SAA Oni T. An interferon-inducible neutrophil-driven blood transcriptional signature in human tuberculosis. Nature (2010) 466:973–77. 10.1038/nature09247 20725040 PMC3492754

[100] Maertzdorf J Repsilber D Parida SK Stanley K Roberts T Black G. Human gene expression profiles of susceptibility and resistance in tuberculosis. Genes Immun. (2011) 12:15–22. 10.1038/gene.2010.51 20861863

[101] Maertzdorf J Ota M Repsilber D Mollenkopf HJ Weiner J Hill PC. Functional correlations of pathogenesis-driven gene expression signatures in tuberculosis. PLoS ONE (2011) 6:e26938. 10.1371/journal.pone.0026938 22046420 PMC3203931

[102] Lesho E Forestiero FJ Hirata MH Hirata RD Cecon L Melo FF. Transcriptional responses of host peripheral blood cells to tuberculosis infection. Tuberculosis (2011) 91:390–9. 10.1016/j.tube.2011.07.002 21835698

[103] Bloom CI Graham CM Berry MPR Wilkinson KA Oni T Rozakeas F. Detectable changes in the blood transcriptome are present after two weeks of antituberculosis therapy. PLoS ONE (2012) 7:e46191. 10.1371/journal.pone.0046191 23056259 PMC3462772

[104] Ottenhoff THM Dass RH Yang N Zhang MM Wong HEE Sahiratmadja E. Genome-wide expression profiling identifies type 1 interferon response pathways in active tuberculosis. PLoS ONE (2012) 7:e45839. 10.1371/journal.pone.0045839 23029268 PMC3448682

[105] Maertzdorf J Weiner J III Mollenkopf H-J Network T Bauer T Prasse A. Common patterns and disease-related signatures in tuberculosis and sarcoidosis. Proc Natl Acad Sci USA. (2012) 109:7853–8. 10.1073/pnas.1121072109 22547807 PMC3356621

[106] Cliff JM Lee J-S Constantinou N Cho J-E Clark TG Ronacher K. Distinct phases of blood gene expression pattern through tuberculosis treatment reflect modulation of the humoral immune response. J Infect Dis. (2012) 207:18–29. 10.1093/infdis/jis499 22872737

[107] Bloom CI Graham CM Berry MPR Rozakeas F Redford PS Wang Y. Transcriptional blood signatures distinguish pulmonary tuberculosis, pulmonary sarcoidosis, pneumonias and lung cancers. PLoS ONE (2013) 8:e70630. 10.1371/journal.pone.0070630 23940611 PMC3734176

[108] Kaforou M Wright VJ Oni T French N Anderson ST Bangani N. Detection of tuberculosis in HIV-infected and -uninfected African adults using whole blood RNA expression signatures: a case-control study. PLoS Med. (2013) 10:e1001538. 10.1371/journal.pmed.1001538 24167453 PMC3805485

[109] Cai Y Yang Q Tang Y Zhang M Liu H Zhang G. Increased complement C1q level marks active disease in human tuberculosis. PLoS ONE (2014) 9:e92340. 10.1371/journal.pone.0092340 24647646 PMC3960215

[110] Anderson ST Kaforou M Brent AJ Wright VJ Banwell CM Chagaluka G. Diagnosis of childhood tuberculosis and host RNA expression in Africa. N Engl J Med. (2014) 370:1712–23. 10.1056/NEJMoa1303657 24785206 PMC4069985

[111] Jacobsen M Repsilber D Gutschmidt A Neher A Feldmann K Mollenkopf HJ. Candidate biomarkers for discrimination between infection and disease caused by *Mycobacterium tuberculosis*. J Mol Med. (2007) 85:613–21. 10.1007/s00109-007-0157-6 17318616

[112] Thompson EG Du Y Malherbe ST Shankar S Braun J Valvo J. Host blood RNA signatures predict the outcome of tuberculosis treatment. Tuberculosis (2017) 107:48–58. 10.1016/j.tube.2017.08.004 29050771 PMC5658513

[113] Suliman S Thompson E Sutherland J Weiner Rd J Ota MOC Shankar S Four-gene Pan-African blood signature predicts progression to tuberculosis. Am J Respir Crit Care Med. (2018) 197:1198–208. 10.1164/rccm.201711-2340OC PMC6019933 29624071

[114] Zak DE Penn-Nicholson A Scriba TJ Thompson E Suliman S Amon LM. A blood RNA signature for tuberculosis disease risk: a prospective cohort study. Lancet (2016) 387:2312–22. 10.1016/S0140-6736(15)01316-1 27017310 PMC5392204

[115] Sloot R Schim van der Loeff MF van Zwet EW Haks MC Keizer ST Scholing M. Biomarkers can identify pulmonary tuberculosis in HIV-infected drug users months prior to clinical diagnosis. EBio Med. (2015) 2:172–9. 10.1016/j.ebiom.2014.12.001 26137541 PMC4484511

[116] Montoya D Inkeles MS Liu PT Realegeno S Teles RMB Vaidya P. IL-32 is a molecular marker of a host defense network in human tuberculosis. Sci Transl Med. (2014) 6:250ra114. 10.1126/scitranslmed.3009546 25143364 PMC4175914

[117] Blankley S Graham CM Levin J Turner J Berry MPR Bloom CI. A 380-gene meta-signature of active tuberculosis compared with healthy controls. Eur Respir J. (2016) 47:1873–6. 10.1183/13993003.02121-2015 27076596 PMC4892351

[118] Liu Y Li H Xiao T Lu Q. Epigenetics in immune-mediated pulmonary diseases. Clin Rev Allergy Immunol. (2013) 45:314–30. 10.1007/s12016-013-8398-3 24242359

[119] Esterhuyse MM Weiner J Caron E Loxton AG Iannaccone M Wagman C. Epigenetics and proteomics join transcriptomics in the quest for tuberculosis biomarkers. MBio (2015) 6:e01187–01115. 10.1128/mBio.01187-15 26374119 PMC4600108

[120] Arts RJW Carvalho A La Rocca C Palma C Rodrigues F Silvestre R. Immunometabolic pathways in BCG-induced trained immunity. Cell Rep. (2016) 17:2562–71. 10.1016/j.celrep.2016.11.011 27926861 PMC5177620

[121] Chen Y-C Chao T-Y Leung S-Y Chen C-J Wu C-C Fang W-F. Histone H3K14 hypoacetylation and H3K27 hypermethylation along with HDAC1 up-regulation and KDM6B down-regulation are associated with active pulmonary tuberculosis disease. Am J Transl Res. (2017) 9:1943–55. 28469799 PMC5411942

[122] Qiao Y Giannopoulou EG Chan CH Park S-H Gong S Chen J. Synergistic activation of inflammatory cytokine genes by interferon-γ-induced chromatin remodeling and toll-like receptor signaling. Immunity (2013) 39:454–69. 10.1016/j.immuni.2013.08.009 24012417 PMC3857147

[123] Ghisletti S Barozzi I Mietton F Polletti S De Santa F Venturini E. Identification and characterization of enhancers controlling the inflammatory gene expression program in macrophages. Immunity (2010) 32:317–28. 10.1016/j.immuni.2010.02.008 20206554

[124] Ostuni R Piccolo V Barozzi I Polletti S Termanini A Bonifacio S. Latent enhancers activated by stimulation in differentiated cells. Cell (2013) 152:157–71. 10.1016/j.cell.2012.12.018 23332752

[125] Kaikkonen MU Spann NJ Heinz S Romanoski CE Allison KA Stender JD. Remodeling of the enhancer landscape during macrophage activation is coupled to enhancer transcription. Mol Cell (2013) 51:310–25. 10.1016/j.molcel.2013.07.010 23932714 PMC3779836

[126] Pacis A Tailleux L Morin AM Lambourne J MacIsaac JL Yotova V. Bacterial infection remodels the DNA methylation landscape of human dendritic cells. Genome Res. (2015) 25:1801–11. 10.1101/gr.192005.115 26392366 PMC4665002

[127] Barreiro LB Tailleux L Pai AA Gicquel B Marioni JC Gilad Y. Deciphering the genetic architecture of variation in the immune response to *Mycobacterium tuberculosis* infection. Proc Natl Acad Sci USA. (2012) 109:1204–9. 10.1073/pnas.1115761109 22233810 PMC3268270

[128] Moores RC Brilha S Schutgens F Elkington PT Friedland JS. Epigenetic Regulation of Matrix Metalloproteinase-1 and−3 Expression in *Mycobacterium tuberculosis* Infection. Front Immunol. (2017) 8:602. 10.3389/fimmu.2017.00602 28596772 PMC5442172

[129] Mehta MD Liu PT microRNAs in mycobacterial disease: friend or foe? Front Genet. (2014) 5:231 10.3389/fgene.2014.00231 25076967 PMC4097432

[130] Wu J Lu C Diao N Zhang S Wang S Wang F. Analysis of microRNA expression profiling identifies miR-155 and miR-155^*^ as potential diagnostic markers for active tuberculosis: a preliminary study. Hum Immunol. (2012) 73:31–7. 10.1016/j.humimm.2011.10.003 22037148

[131] Wang C Yang S Sun G Tang X Lu S Neyrolles O. Comparative miRNA expression profiles in individuals with latent and active tuberculosis. PLoS ONE (2011) 6:e25832. 10.1371/journal.pone.0025832 22003408 PMC3189221

[132] Abd-El-Fattah AA Sadik NAH Shaker OG Aboulftouh ML. Differential MicroRNAs expression in serum of patients with lung cancer, pulmonary tuberculosis, and pneumonia. Cell Biochem Biophys. (2013) 67:875–84. 10.1007/s12013-013-9575-y 23559272

[133] Fu Y Yi Z Wu X Li J Xu F. Circulating microRNAs in patients with active pulmonary tuberculosis. J Clin Microbiol. (2011) 49:4246–51. 10.1128/JCM.05459-11 21998423 PMC3232949

[134] Spinelli SV Diaz A D'Attilio L Marchesini MM Bogue C Bay ML. Altered microRNA expression levels in mononuclear cells of patients with pulmonary and pleural tuberculosis and their relation with components of the immune response. Mol Immunol. (2013) 53:265–9. 10.1016/j.molimm.2012.08.008 22964481

[135] Yi Z Fu Y Ji R Li R Guan Z. Altered microRNA signatures in sputum of patients with active pulmonary tuberculosis. PLoS ONE (2012) 7:e43184. 10.1371/journal.pone.0043184 22900099 PMC3416796

[136] Kleinsteuber K Heesch K Schattling S Kohns M Sander-Jülch C Walzl G. Decreased expression of miR-21, miR-26a, miR-29a, and miR-142-3p in CD4^+^ T cells and peripheral blood from tuberculosis patients. PLoS ONE (2013) 8:e61609. 10.1371/journal.pone.0061609 23613882 PMC3628900

[137] van Rensburg IC du Toit L Walzl G du Plessis N Loxton AG. Decreased neutrophil–associated miRNA and increased B-cell associated miRNA expression during tuberculosis. Gene (2018) 655:35–41. 10.1016/j.gene.2018.02.052 29477867

[138] Dorhoi A Iannaccone M Farinacci M Faé KC Schreiber J Moura-Alves P. MicroRNA-223 controls susceptibility to tuberculosis by regulating lung neutrophil recruitment. J Clin Invest. (2013) 123:4836–48. 10.1172/JCI67604 24084739 PMC3809781

[139] Wang J Yang K Zhou L Minhaowu Wu Y Zhu M. MicroRNA-155 promotes autophagy to eliminate intracellular mycobacteria by targeting Rheb. PLoS Pathog. (2013) 9:e1003697. 10.1371/journal.ppat.1003697 24130493 PMC3795043

[140] Rothchild AC Sissons JR Shafiani S Plaisier C Min D Mai D. MiR-155–regulated molecular network orchestrates cell fate in the innate and adaptive immune response to *Mycobacterium tuberculosis*. Proc Natl Acad Sci USA. (2016) 113:E6172–81. 10.1073/pnas.1608255113 27681624 PMC5068277

[141] Iwai H Funatogawa K Matsumura K Kato-Miyazawa M Kirikae F Kiga K. MicroRNA-155 knockout mice are susceptible to *Mycobacterium tuberculosis* infection. Tuberculosis (2015) 95:246–50. 10.1016/j.tube.2015.03.006 25846955

[142] Li D Li D Wang T Song X Qucuo M Yang B. Genetic study of two single nucleotide polymorphisms within corresponding microRNAs and susceptibility to tuberculosis in a Chinese Tibetan and Han population. Hum Immunol. (2011) 72:598–602. 10.1016/j.humimm.2011.03.004 21524676

[143] Wu H Wang Y Zhang Y Yang M Lv J Liu J. TALE nickase-mediated SP110 knockin endows cattle with increased resistance to tuberculosis. Proc Natl Acad Sci USA. (2015) 112:E1530–9. 10.1073/pnas.1421587112 25733846 PMC4386332

[144] Pan H Yan BS Rojas M Shebzukhov YV Zhou H Kobzik L. *Ipr1* gene mediates innate immunity to tuberculosis. Nature (2005) 434:767–72. 10.1038/nature03419 15815631 PMC1388092

[145] Tosh K Campbell SJ Fielding K Sillah J Bah B Gustafson P. Variants in the *SP110* gene are associated with genetic susceptibility to tuberculosis in West Africa. Proc Natl Acad Sci USA. (2006) 103:10364–8. 10.1073/pnas.0603340103 16803959 PMC1502463

[146] Babb C Keet EH van Helden PD Hoal EG *SP110* polymorphisms are not associated with pulmonary tuberculosis in a South African population. Hum Genet. (2007) 121:521–2. 10.1007/s00439-007-0335-1 17287948

[147] Thye T Browne EN Chinbuah MA Gyapong J Osei I Owusu-Dabo E. No associations of human pulmonary tuberculosis with *Sp110* variants. J Med Genet. (2006) 43:e32. 10.1136/jmg.2005.037960 16816019 PMC2564561

[148] Zhou Y Tan C-Y Mo Z-J Gao Q He D Li J. Polymorphisms in the *SP110* and TNF-α gene and susceptibility to pulmonary and spinal tuberculosis among Southern Chinese Population. Dis Markers (2017) 2017:4590235. 10.1155/2017/4590235 29430075 PMC5752994

[149] Png E Alisjahbana B Sahiratmadja E Marzuki S Nelwan R Adnan I Polymorphisms in *SP110* are not associated with pulmonary tuberculosis in Indonesians. Infect Genet Evol. (2012) 12:1319–23. 10.1016/j.meegid.2012.04.006 22522001

[150] Casanova J-L Abel L. The genetic theory of infectious diseases: a brief history and selected illustrations. Annu Rev Genomics Hum Genet. (2013) 14:215–43. 10.1146/annurev-genom-091212-153448 23724903 PMC4980761

[151] Gagneux S Deriemer K Van T Kato-Maeda M de Jong BC Narayanan S. Variable host-pathogen compatibility in *Mycobacterium tuberculosis*. Proc Natl Acad Sci USA. (2006) 103:2869–73. 10.1073/pnas.0511240103 16477032 PMC1413851

[152] Salie M van der Merwe L Möller M Daya M van der Spuy GD van Helden PD. Associations between human leukocyte antigen class I variants and the *Mycobacterium tuberculosis* subtypes causing disease. J Infect Dis. (2014) 209:216–23. 10.1093/infdis/jit443 23945374 PMC3873786

[153] Velez DR Hulme WF Myers JL Weinberg JB Levesque MC Stryjewski ME. *NOS2A, TLR4*, and *IFNGR1* interactions influence pulmonary tuberculosis susceptibility in African-Americans. Hum Genet. (2009) 126:643–53. 10.1007/s00439-009-0713-y 19575238 PMC2881538

[154] Velez DR Hulme WF Myers JL Stryjewski ME Abbate E Estevan R. Association of *SLC11A1* with tuberculosis and interactions with *NOS2A* and *TLR2* in African-Americans and Caucasians. Int J Tuberc Lung Dis. (2009) 13:1068–76. 19723394 PMC2902362

[155] Caws M Thwaites G Dunstan S Hawn TR Lan NT Thuong NT. The influence of host and bacterial genotype on the development of disseminated disease with *Mycobacterium tuberculosis*. PLoS Pathog. (2008) 4:e1000034. 10.1371/journal.ppat.1000034 18369480 PMC2268004

[156] Naranbhai V. The role of host genetics (and genomics) in tuberculosis. Microbiol Spectr. (2016) 4. 10.1128/microbiolspec.TBTB2-0011-2016 27787193

